# GALNT2 expression is associated with glucose control and serum metabolites in patients with type 2 diabetes

**DOI:** 10.1007/s00592-024-02280-7

**Published:** 2024-04-17

**Authors:** Vincenzo Trischitta, Alessandra Antonucci, Jerzy Adamski, Cornelia Prehn, Claudia Menzaghi, Antonella Marucci, Rosa Di Paola

**Affiliations:** 1 Research Unit of Diabetes and Endocrine Diseases, Fondazione IRCCS Casa Sollievo Della Sofferenza, 71013 San Giovanni Rotondo, Foggia Italy; 2 Department of Experimental Medicine, Sapienza University, 00161 Rome, Italy; 3 Present Address: Department of Systems Medicine, University of Rome Tor Vergata, Rome, Italy; 4 Institute of Experimental Genetics, Helmholtz Zentrum München, German Research Center for Environmental Health, Ingolstädter Landstraße 1, 85764 Neuherberg, Germany; 5 Department of Biochemistry, Yong Loo Lin School of Medicine, National University of Singapore, 8 Medical Drive, Singapore, 117597 Singapore; 6 Institute of Biochemistry, Faculty of Medicine, University of Ljubljana, Vrazov trg 2, 1000 Ljubljana, Slovenia

**Keywords:** *GALNT2*, Glycine, Phenylethylamine, Taurine, Insulin resistance metabolic abnormalities, Metabolic signature

## Abstract

**Aims:**

Aim of this study was to investigate in type 2 diabetes whether expression level of *GALNT2*, a positive modulator of insulin sensitivity, is associated with a metabolic signature.

**Methods:**

Five different metabolite families, including acylcarnitines, aminoacids, biogenic amines, phospholipids and sphingolipids were investigated in fasting serum of 70 patients with type 2 diabetes, by targeted metabolomics. *GALNT2* expression levels were measured in peripheral white blood cells by RT-PCR. The association between *GALNT2* expression and serum metabolites was assessed using false discovery rate followed by stepwise selection and, finally, multivariate model including several clinical parameters as confounders. The association between *GALNT2* expression and the same clinical parameters was also investigated.

**Results:**

*GALNT2* expression was independently correlated with HbA1c levels (*P* value = 0.0052), a finding that is the likely consequence of the role of *GALNT2* on insulin sensitivity. *GALNT2* expression was also independently associated with serum levels of the aminoacid glycine (*P* value = 0.014) and two biogenic amines phenylethylamine (*P* value = 0.0065) and taurine (*P* value = 0.0011). The association of *GALNT2* expression with HbA1c was not mediated by these three metabolites.

**Conclusions:**

Our data indicate that in type 2 diabetes the expression of *GALNT2* is associated with several serum metabolites. This association needs to be further investigated to understand in depth its role in mediating the effect of GALNT2 on insulin sensitivity, glucose control and other clinical features in people with diabetes.

**Supplementary Information:**

The online version contains supplementary material available at 10.1007/s00592-024-02280-7.

## Introduction

Insulin resistance is a common pathogenic ground for many highly prevalent diseases. These include atherogenic dyslipidemia [1], type 2 diabetes, obesity, hypertension [1], and closely related cardiovascular disease and renal dysfunction [1, 2], all major causes of morbidity and mortality worldwide [3]. Unraveling the intimate molecular signature of insulin signaling would contribute to understanding the pathogenesis of all the above-mentioned diseases and is therefore urgently needed.

In the last few years several evidences suggested that *GALNT2* (coding for ppGalNAc-T2, involved in the initiation step of O-linked glycosylation [4, 5]), modulates insulin sensitivity [6]. In fact, in cultured human liver cells (HepG2) *GALNT2* down-regulation reduces insulin-induced insulin receptor, IRS-1 and protein kinase beta Akt2 phosphorylation, as well as expression of gluconeogenic enzyme phosphoenolpyruvate carboxykinase (PEPCK) [7]. Also, GALNT2 over-expression in mouse pre-adipocytes fibroblasts (3T3L1) stimulates adipocyte maturation and enlargement, through increasing insulin signaling [8]. Although, the biological mechanism through which GALNT2 affects insulin signaling is still unknown, the inverse correlation between *GALNT2* and *ENPP1* (a negative modulator of insulin signaling) expression, suggests that *ENPP1* downregulation mediates, at least in part, the effect of *GALNT2* on insulin sensitivity [9–14].

In addition, several data from both humans and animal models consistently demonstrated the contribution of GALNT2 to several highly prevalent metabolic abnormalities related to insulin resistance, namely atherogenic dyslipidemia [6, 15–23], type 2 diabetes [24, 25], obesity [6, 17, 26] and polycystic ovary syndrome [27]. Unfortunately, the exact biological mechanisms through which *GALNT2* affect insulin signaling is not completely understood [6].

Thanks to the recent advances in bioinformatics and technology, measuring hundreds or thousands of metabolites in biological samples has unraveled specific signatures related to altered metabolic states, including insulin resistance, type 2 diabetes and obesity [28].

We investigated whether *GALNT2* expression is characterized by a specific metabolic signature. In details, five different metabolite families were investigated, including acylcarnitines, aminoacids, biogenic amines, phospholipids and sphingolipids.

## Research design and methods

### Participants

The study cohort consisted of 70 patients with type 2 diabetes (according to the American Diabetes Association 2003 criteria), belonging to the Gargano Mortality Study 2 (GMS, [29]) including individuals recruited from 2008 to 2010 at the Endocrine Unit of Fondazione Istituto di Ricovero e Cura a Carattere Scientifico “Casa Sollievo della Sofferenza” in San Giovanni Rotondo. Our 70 study patients were randomly selected among those whose RNA sample at recruitment was available. The study protocols and the informed consent procedures were approved by the local Institutional Ethic Committee.

### Metabolite quantification and normalization

Metabolites were measured in baseline fasting serum at the Genome Analysis Center, Helmholtz Zentrum München with a targeted metabolomics approach by Absolute*IDQ* p180 Kit (Biocrates Life Sciences AG, Innsbruck, Austria), as previously described [29]. The assay includes free carnitine, 40 acylcarnitines (Cx:y), 21 amino acids (19 proteinogenic + citrulline + ornithine), 21 biogenic amines, hexoses (sum of hexoses: ∼ 90 to 95% glucose), 90 glycerophospholipids (14 lysophosphatidylcholines [lysoPC] and 76 phosphatidylcholines [PC], and 15 sphingolipids [SMx:y]). Three quality control samples (sex-mixed human plasma provided by the manufacturer) and one zero sample (PBS) were included in each randomized plate.

### GALNT2 expression levels

Gene expression levels were measured in peripheral white blood cells by using Gene Expression Assay on Demand Kit Reagents (Applera Life Technologies, Carlsbad, CA), by means of RT-PCR as previously described [24]. Expression levels of *GALNT2* were calculated by using the comparative DCT method normalizing, the amount of *GALNT2* was normalized to GAPDH, B actin and 18S considered together (geometric mean) [30] and related to a control RNA as calibrator (2^−ΔΔCT^).

### Statistical analysis

Patients’ baseline characteristics are reported as mean ± SD, or median and interquartile range for continuous skewed variables (|skewness| > 1) and frequency and percentage for categorical variables. Values of serum metabolites below the limit of detection have been replaced by the limit of detection itself.

For pre-processing of data, normal distribution and skewness were tested in all metabolites and covariates. Since the metabolites’ distributions were skewed, all concentrations were log_2_ transformed and standardized. The 2^−ΔΔCT^ data of *GALNT2* expression levels were also standardized. Clinical parameters with percentage of missing value less than 5% (i.e., BMI 1.4% and HbA1c 2.8%) were imputed with random forest method [31].

The association between *GALNT2* expression and serum metabolites within each metabolite family (i.e., acylcarnitine, amino acids, biogenic amines, glycerophospholipids and sphingolipids), was firstly assessed in a univariate model by using false discovery rate (FDR) to take into account multiple comparisons and then in a multivariate model including age, sex, smoking habits, BMI, HbA1c, diabetes duration, eGFR, and anti-hypertensive, anti-hyperglycemia and lipid-lowering therapies. Finally, in order to minimize potential multicollinearity issues, metabolites that were independently associated with *GALNT2* expression, entered jointly a stepwise selection (SSE criterion: *p *value of the F-statistic to enter and to remove term to the model less than 0.05 and greater than 0.10 respectively). A *P* value < 0.05 was considered statistically significant.

All analyses were performed using SAS 9.4 (SAS Institute, Cary, NC) and Matlab R2022—Statistics and Machine Learning Toolbox (The MathWorks, Inc., Natick, MA).

## Results

### Study patients

Clinical features as well as diabetes duration and ongoing treatments of the 70 study participants with type 2 diabetes are reported in Table 1.

**Table 1:** Clinical characteristics of study patients (n = 70)

Women (%)	28.6
Age (years)	55.3 ± 10.1
Current smokers (%)	24.3
Diabetes duration (years)	11 ± 7.8
BMI (kg/m^2^)	30.5 ± 5.4
HbA1c (%)	7.7 ± 2.1
eGFR (mL/min/1.73 m^2^)	101.2 (74.5–109.6)
HDL-cholesterol (mg/dL)	43.2 ± 11.4
Triglycerides (mg/dL)	125.5 (88–173)
Anti-hypertensive therapy (%)	72.9
Anti-hyperglycemia therapy	
Diet (%)	4.3
OA (%)	42.8
Insulin ± OA (%)	52.9
Lipid-lowering therapy	
None (%)	21.4
Statins (%)	75.7
Others (%)	2.9

Continuous variables were reported as mean ± SD or as median (interquartile range) for skewed variables (skewness > |± 1|), whereas categorical variables as total frequencies

BMI, Body Mass Index; HbA1c, glycated hemoglobin; eGFR, estimated glomerular filtration rate as CKD-EPI formula [51]; HDL, high density lipoprotein; OA, Oral agents

In univariate analyses, *GALNT2* expression levels were correlated with HbA1c (β ± SE = − 0.198 ± 0.069; *P* value = 0.0052) and BMI (β ± SE = − 0.045 ± 0.22; *P* value = 0.044) but not with other features (Table 2). Only the association with HbA1c remained significant in a fully adjusted model comprising all available clinical information (Table 2).

**Table 2:** Association between GALNT2 expression levels and patients’ clinical features

	β ± SE	*P* value	*P* value*
Age (years)	− 0.001 ± 0.012	0.95	0.89
Gender	0.36 ± 0.26	0.18	0.29
Current smokers (%)	− 0.41 ± 0.28	0.15	0.17
BMI (kg/m^2^)	− 0.045 ± 0.02	*0.044*	0.15
Diabetes duration (years)	− 0.011 ± 0.016	0.47	0.79
HbA1c (%)	− 0.198 ± 0.069	*0.0052*	*0.026*
eGFR (mL/min/1.73 m^2^)	0.009 ± 0.005	0.061	0.36
Anti-hypertensive therapy (%)	− 0.329 ± 0.268	0.22	0.24
Anti-hyperglycemia therapy	− 0.287 ± 0.205	0.17	0.89
Lipid-lowering therapy	− 0.319 ± 0.261	0.23	0.38

BMI, Body Mass Index; HbA1c, glycated hemoglobin; eGFR, estimated glomerular filtration rate as CKD-EPI formula [51]

*P value in a multivariate model including all variables listed in the table

Italic font indicate a nominal statistical significance

### GALNT2 and metabolites

Five out of 188 metabolites measured (i.e., carnosine, DOPA, dopamine, nitrotyrosine, *cis*-4-Hydroxyproline) were excluded from the analyses because their value was below the detection limit in > 80% samples.

**Fig. 1:**
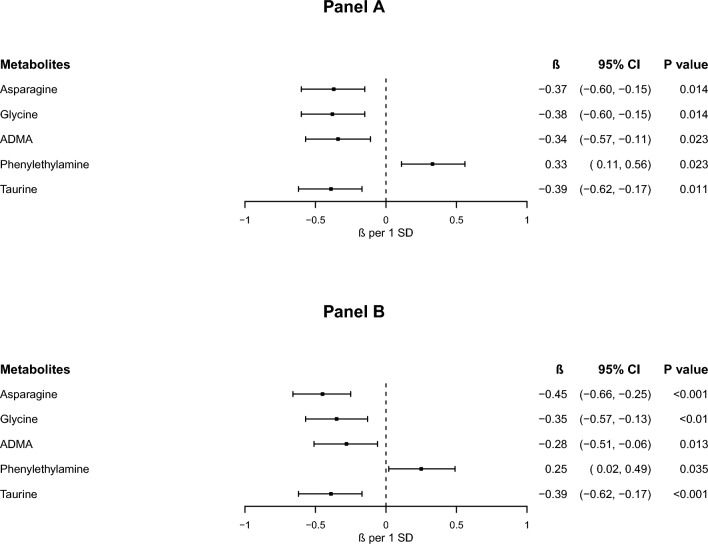
Associations between GALNT2 expression levels and serum metabolites. β (per 1 SD increase *GALNT2* expression) and 95% CIs were estimated in univariate (**A**) and in multivariate (**B**) regression models, adjusting for age at recruitment, sex, smoking habit, BMI, HbA1c, eGFR, diabetes duration, and ongoing treatment

Correlation between *GALNT2* expression and metabolites was investigated separately in the five metabolite families. After adjusting for multiple comparisons with false discovery rate (FDR) procedure [32], *GALNT2* was associated with two aminoacids (asparagine and glycine: β ± SE = − 0.37 ± 0.11 and − 0.38 ± 0.11, *P* values = 0.014 for both) and three biogenic amines (ADMA, phenylethylamine and taurine: β ± SE = − 0.34 ± 0.11, 0.33 ± 0.11 and − 0.39 ± 0.11, *P* values being 0.023, 0.023 and 0.011, respectively) (Fig. 1A). Conversely, no associations with acylcarnitines, phospholipids and sphingolipids were observed (Supplementary Table 1).

All five metabolites remained significantly associated with *GALNT2* mRNA levels in a multivariate model that included age, gender, smoking habits, BMI, HbA1c, diabetes duration, eGFR, and current treatments (*P* values = 0.0027, 0.000049, 0.013253, 0.035 and 0.00067, for glycine, asparagine, ADMA, phenylethylamine and taurine, respectively), thus indicating that their correlation with *GALNT2* expression is independent of the most important clinical variables (Fig. 1B).

After a stepwise (forward–backward) analyses aimed at taking into account the correlations among metabolites from the same family, the aminoacid glycine (*P* value = 0.0014) and two biogenic amines phenylethylamine (*P* value = 0.0065) and taurine (*P* value = 0.0011) remained associated with *GALNT2* expression levels.

Finally, none of the three metabolites correlated with HbA1c (data not shown) nor influenced the observed correlation between HbA1c and *GALNT2* expression (β ± SE and *P* values moving to − 0.190 ± 0.064, *P* value = 0.004; − 0.165 ± 0.067; *P* value = 0.017 and − 0.203 ± 0.063; *P* value = 0.0018 after adjusting for glycine, phenylethylamine and taurine, respectively).

## Discussion

Our study investigated whether in patients with type 2 diabetes *GALNT2* expression is characterized by a specific signature belonging to several metabolite families, including acylcarnitines, amino acids, biogenic amines, glycerophospholipids and sphingolipids. We also investigated the association between *GALNT2* expression and several clinical variables. Firstly, *GALNT2* expression was independently and negatively correlated with HbA1c levels, a finding that may well be secondary to the reported positive effect of *GALNT2* on insulin sensitivity [6]. This link is also suggested by the negative association between *GALNT2* and BMI, which however did not survive a multivariable model comprising several additional clinical variables. The expression of *GALNT2* was also independently associated with serum levels of the aminoacid glycine and arginine and the biogenic amines phenylethylamine, taurine and ADMA. When collinearity within the same metabolite family was taken into account, only glycine, taurine and phenethylamine remained associated with GALNT2 expression levels. Interestingly, the association between *GALNT2* expression and HbA1c was not modified taking into account these three latter metabolites, thus suggesting they do not mediate the positive effect of *GALNT2* on glucose control.

Previous studies have highlighted that glycine, is consistently and negatively associated with reduced insulin sensitivity [33, 34], impaired glucose homeostasis [33, 35–39] and liver steatosis [38]. In addition, low glycine levels have been reported to predict prospectively the development of type 2 diabetes [36, 37, 39–41]. Also plasma taurine is reduced in subjects with metabolic syndrome [42], diabetes [43, 44] and obesity [45, 46] as well as in obese animals [47]. All these previous evidences on the role of glycine and taurine make our present correlative findings compatible with the belief that *GALNT2* is involved in insulin sensitivity and resistance [6]. On the other hand, we acknowledge that their interpretation is not straightforward. Indeed, if the positive effect of *GALNT2* on insulin sensitivity were mediated by the above-mentioned metabolites, one would expect an association with glycine and taurine in the opposite direction to that observed in our study (i.e., positive rather than negative correlation). This makes unlikely that glycine or taurine mediate the positive effect of *GALNT2* on glucose control as also suggested by the observation that the association between *GALNT2* and HbA1c does not change much after adjusting for these two metabolites. It can therefore be hypothesized that the counterintuitive associations we here report represent a homeostatic mechanism in which *GALNT2* upregulation acts as a fine tuner to counteract insulin-resistance induced (or simply marked) by low levels of glycine and taurine. Conversely, no published data are available on circulating phenethylamine levels in different conditions related to metabolic abnormalities. Interestingly, fecal phenethylamine levels, derived from bacterial fermentation of amino acids in the gut, are correlated positively with glucose intolerance and negatively with improved diet-induced insulin sensitivity [48] while urinary phenylethylamine levels were higher in obese women as compared to their normal/underweight counterparts [49]. These reports suggest that phenethylamine also plays a role in several clinically relevant insulin resistance phenotypes. Unfortunately, it is not known whether and how fecal and urinary phenethylamine levels are correlated with serum levels, thus making difficult the interpretation of the positive association we observed between *GALNT2* expression and circulating phenethylamine. In all, we do acknowledge that the associations of GALNT2 expression levels with HbA1c and several circulating metabolites may imply more than a single and unambiguous interpretation and, consequently, does not allow, yet, to define a clear metabolic signature linking GALNT2, circulating metabolites and clinical features related to insulin resistance.

Among limitation of our study, we do recognize that expression data in peripheral white blood cells may not mirror those of other tissues, including the most important ones for glucose homeostasis maintenance. On the other hand, this cell model has been successfully used in *cis*-eQTL, *trans*-eQTL analyses from the eQTLGen consortium (https://www.eqtlgen.org/) aimed at understanding the genetic architecture underlying complex traits including insulin resistance-related abnormalities [50]. Furthermore, we recognize that the small sample size of our study impacts statistical power, thus making it possible that we missed additional associations between *GALNT2* expression and circulating levels of other metabolites as well as subtle effects of the associated metabolites on the role of *GALNT2* on HbA1c and other clinical features (i.e., false-negative results).

In conclusion, our data indicate for the first time that in type 2 diabetes the expression of *GALNT2* is associated with several serum metabolites. This association needs to be further investigated. To understand in depth its role in mediating the effect of GALNT2 on insulin sensitivity, glucose control and other clinical features in people with diabetes. If our current findings are confirmed and deepened by other studies to gain a better comprehension of the molecular effects of GALNT2 on insulin sensitivity, this will likely become instrumental in the discovery of hitherto unknown pathogenic nodes that can be targeted with new therapies in patients with insulin resistance and related anomalies.

### Supplementary Information

Below is the link to the electronic supplementary material.

Supplementary file1 (DOCX 36 kb)

## Data Availability

Data are available upon reasonable request.
