# Correction: GALNT2 expression is associated with glucose control and serum metabolites in patients with type 2 diabetes

**DOI:** 10.1007/s00592-024-02346-6

**Published:** 2024-08-08

**Authors:** Vincenzo Trischitta, Alessandra Antonucci, Jerzy Adamski, Cornelia Prehn, Claudia Menzaghi, Antonella Marucci, Rosa Di Paola

**Affiliations:** 1 Research Unit of Diabetes and Endocrine Diseases, Fondazione IRCCS Casa Sollievo Della Sofferenza, San Giovanni Rotondo (FG), 71013 Foggia, Italy; 2 Department of Experimental Medicine, Sapienza University, Rome, 00161 Italy; 3 Present Address: Department of Systems Medicine, University of Rome Tor Vergata, Rome, Italy; 4 Institute of Experimental Genetics, Helmholtz Zentrum München, German Research Center for Environmental Health, Ingolstädter Landstraße 1, 85764, Neuherberg, Germany; 5 Department of Biochemistry, Yong Loo Lin School of Medicine, National University of Singapore, 8 Medical Drive, Singapore, 117597 Singapore; 6 Institute of Biochemistry, Faculty of Medicine, University of Ljubljana, Vrazov trg 2, 1000, Ljubljana, Slovenia

**Correction: Acta Diabetologica**

10.1007/s00592-024-02280-7

In this article, the author names Jerzy Adamski and Cornelia Prehn were missing from the author list.

The original article has been corrected.

