# Supplementary material for: GALNT2 expression is associated with glucose control and serum metabolites in patients with type 2 diabetes
Source: Acta Diabetol. 2024 Apr 17;61(8):1007–13. doi: 10.1007/s00592-024-02280-7 (PMC11329529; doi:10.1007/s00592-024-02280-7)
Supplement: Supplementary file 1 — Supplementary file1 (DOCX 36 kb) [file 592_2024_2280_MOESM1_ESM.docx]

**Supplemental Material**

**Supplemental Table 1: Association between GALNT2 expression levels and serum metabolite.**

|  | **Metabolite** | **Estimate** | ***P* value (unadj)** | **P value (FDR)** |
| --- | --- | --- | --- | --- |
| **'Acylcarnitines'** | 'C0' | -0.178 | 1.37E-01 | 4.60E-01 |
|  | 'C2' | -0.056 | 6.40E-01 | 8.93E-01 |
|  | 'C3' | -0.162 | 1.77E-01 | 5.07E-01 |
|  | 'C3_DC_C4_OH' | -0.102 | 3.97E-01 | 8.82E-01 |
|  | 'C3_OH' | -0.035 | 7.70E-01 | 8.98E-01 |
|  | 'C3_1' | -0.075 | 5.37E-01 | 8.93E-01 |
|  | 'C4' | -0.197 | 9.92E-02 | 4.60E-01 |
|  | 'C4_1' | -0.217 | 6.85E-02 | 4.60E-01 |
|  | 'C5' | -0.243 | 4.08E-02 | 4.60E-01 |
|  | 'C5_DC_C6_OH' | -0.244 | 4.06E-02 | 4.60E-01 |
|  | 'C5_M_DC' | -0.07 | 5.61E-01 | 8.93E-01 |
|  | 'C5_OH_C3_DC_M' | -0.082 | 4.99E-01 | 8.93E-01 |
|  | 'C5_1' | -0.175 | 1.44E-01 | 4.60E-01 |
|  | 'C5_1_DC' | -0.038 | 7.54E-01 | 8.98E-01 |
|  | 'C6_C4_1_DC' | -0.202 | 9.13E-02 | 4.60E-01 |
|  | 'C6_1' | 0.06 | 6.20E-01 | 8.93E-01 |
|  | 'C7_DC' | 0.121 | 3.15E-01 | 7.42E-01 |
|  | 'C8' | -0.048 | 6.92E-01 | 8.93E-01 |
|  | 'C9' | 0.057 | 6.37E-01 | 8.93E-01 |
|  | 'C10' | 0.026 | 8.31E-01 | 8.98E-01 |
|  | 'C10_1' | 0.039 | 7.44E-01 | 8.98E-01 |
|  | 'C10_2' | -0.03 | 8.01E-01 | 8.98E-01 |
|  | 'C12' | 0.061 | 6.13E-01 | 8.93E-01 |
|  | 'C12_DC' | 0.18 | 1.33E-01 | 4.60E-01 |
|  | 'C12_1' | 0.048 | 6.91E-01 | 8.93E-01 |
|  | 'C14' | -0.012 | 9.23E-01 | 9.23E-01 |
|  | 'C14_1' | -0.017 | 8.89E-01 | 9.11E-01 |
|  | 'C14_1_OH' | 0.029 | 8.11E-01 | 8.98E-01 |
|  | 'C14_2' | 0.059 | 6.23E-01 | 8.93E-01 |
|  | 'C14_2_OH' | -0.021 | 8.62E-01 | 9.07E-01 |
|  | 'C16' | -0.148 | 2.18E-01 | 5.45E-01 |
|  | 'C16_OH' | -0.197 | 1.00E-01 | 4.60E-01 |
|  | 'C16_1' | -0.178 | 1.38E-01 | 4.60E-01 |
|  | 'C16_1_OH' | -0.063 | 5.99E-01 | 8.93E-01 |
|  | 'C16_2' | -0.048 | 6.89E-01 | 8.93E-01 |
|  | 'C16_2_OH' | -0.199 | 9.59E-02 | 4.60E-01 |
|  | 'C18' | -0.156 | 1.93E-01 | 5.15E-01 |
|  | 'C18_1' | -0.219 | 6.71E-02 | 4.60E-01 |
|  | 'C18_1_OH' | -0.173 | 1.49E-01 | 4.60E-01 |
|  | 'C18_2' | -0.086 | 4.74E-01 | 8.93E-01 |
| **'Amino Acids'** | 'Ala' | -0.213 | 7.44E-02 | 1.20E-01 |
|  | 'Arg' | -0.17 | 1.56E-01 | 2.04E-01 |
|  | 'Asn' | -0.373 | 1.36E-03 | 1.42E-02 |
|  | 'Asp' | -0.279 | *1.85E-02* | *5.55E-02* |
|  | 'Cit' | -0.275 | 2.02E-02 | 5.30E-02 |
|  | 'Gln' | 0.058 | 6.30E-01 | 6.61E-01 |
|  | 'Glu' | -0.174 | 1.47E-01 | 2.06E-01 |
|  | 'Gly' | -0.375 | *1.29E-03* | *2.70E-02* |
|  | 'His' | -0.261 | 2.81E-02 | 6.56E-02 |
|  | 'Ile' | -0.23 | 5.36E-02 | 1.02E-01 |
|  | 'Leu' | -0.218 | 6.72E-02 | 1.18E-01 |
|  | 'Lys' | -0.251 | 3.49E-02 | 7.32E-02 |
|  | 'Met' | -0.058 | 6.29E-01 | 6.94E-01 |
|  | 'Orn' | -0.288 | 1.51E-02 | 6.32E-02 |
|  | 'Phe' | -0.309 | 8.78E-03 | 6.15E-02 |
|  | 'Pro' | -0.169 | 1.59E-01 | 1.96E-01 |
|  | 'Ser' | -0.287 | 1.53E-02 | 5.35E-02 |
|  | 'Thr' | -0.3 | 1.11E-02 | 5.81E-02 |
|  | 'Trp' | -0.056 | 6.45E-01 | 6.45E-01 |
|  | 'Tyr' | -0.101 | 4.04E-01 | 4.71E-01 |
|  | 'Val' | -0.205 | 8.57E-02 | 1.28E-01 |
| **'Biogenic Amines'** | 'Ac_Orn' | 0.04 | 7.42E-01 | 7.42E-01 |
|  | 'ADMA' | -0.339 | *3.78E-03* | *2.34E-02* |
|  | 'alpha_AAA' | -0.112 | 3.55E-01 | 4.36E-01 |
|  | 'Creatinine' | -0.283 | 1.70E-02 | 6.03E-02 |
|  | 'Histamine' | 0.128 | 2.89E-01 | 4.20E-01 |
|  | 'Kynurenine' | -0.264 | 2.64E-02 | 6.03E-02 |
|  | 'Met_SO' | -0.197 | 9.96E-02 | 1.77E-01 |
|  | 'PEA' | 0.334 | *4.39E-03* | *2.34E-02* |
|  | 'Putrescine' | 0.078 | 5.21E-01 | 5.55E-01 |
|  | 'SDMA' | -0.267 | 2.46E-02 | 6.03E-02 |
|  | 'Serotonin' | -0.118 | 3.25E-01 | 4.34E-01 |
|  | 'Spermidine' | -0.084 | 4.85E-01 | 5.54E-01 |
|  | 'Spermine' | -0.204 | 8.85E-02 | 1.77E-01 |
|  | 't4_OH_Pro' | -0.132 | 2.72E-01 | 4.20E-01 |
|  | 'Taurine' | -0.394 | *6.85E-04* | *1.10E-02* |
|  | 'total_DMA' | -0.275 | 2.02E-02 | 6.03E-02 |
| **'Glycerophospholipids'** | 'lysoPC_a_C14_0' | -0.174 | 1.48E-01 | 2.66E-01 |
|  | 'lysoPC_a_C16_0' | -0.321 | 6.41E-03 | 1.59E-01 |
|  | 'lysoPC_a_C16_1' | -0.234 | 4.94E-02 | 1.60E-01 |
|  | 'lysoPC_a_C17_0' | -0.261 | 2.81E-02 | 1.59E-01 |
|  | 'lysoPC_a_C18_0' | -0.3 | 1.11E-02 | 1.59E-01 |
|  | 'lysoPC_a_C18_1' | -0.239 | 4.46E-02 | 1.60E-01 |
|  | 'lysoPC_a_C18_2' | -0.051 | 6.72E-01 | 7.55E-01 |
|  | 'lysoPC_a_C20_3' | -0.252 | 3.43E-02 | 1.59E-01 |
|  | 'lysoPC_a_C20_4' | -0.266 | 2.50E-02 | 1.59E-01 |
|  | 'lysoPC_a_C24_0' | 0.03 | 8.04E-01 | 8.55E-01 |
|  | 'lysoPC_a_C26_0' | -0.229 | 5.44E-02 | 1.60E-01 |
|  | 'lysoPC_a_C26_1' | -0.205 | 8.63E-02 | 1.92E-01 |
|  | 'lysoPC_a_C28_0' | -0.265 | 2.56E-02 | 1.59E-01 |
|  | 'lysoPC_a_C28_1' | -0.198 | 9.75E-02 | 2.04E-01 |
|  | 'PC_aa_C24_0' | -0.275 | 2.02E-02 | 1.59E-01 |
|  | 'PC_aa_C26_0' | -0.137 | 2.56E-01 | 4.04E-01 |
|  | 'PC_aa_C28_1' | -0.09 | 4.56E-01 | 5.79E-01 |
|  | 'PC_aa_C30_0' | -0.057 | 6.39E-01 | 7.28E-01 |
|  | 'PC_aa_C30_2' | -0.143 | 2.33E-01 | 3.82E-01 |
|  | 'PC_aa_C32_0' | -0.116 | 3.34E-01 | 4.77E-01 |
|  | 'PC_aa_C32_1' | -0.107 | 3.73E-01 | 5.10E-01 |
|  | 'PC_aa_C32_2' | -0.017 | 8.86E-01 | 9.13E-01 |
|  | 'PC_aa_C32_3' | -0.065 | 5.90E-01 | 6.86E-01 |
|  | 'PC_aa_C34_1' | -0.19 | 1.12E-01 | 2.24E-01 |
|  | 'PC_aa_C34_2' | -0.119 | 3.22E-01 | 4.67E-01 |
|  | 'PC_aa_C34_3' | -0.082 | 4.96E-01 | 6.04E-01 |
|  | 'PC_aa_C34_4' | -0.102 | 3.97E-01 | 5.25E-01 |
|  | 'PC_aa_C36_0' | -0.165 | 1.69E-01 | 2.93E-01 |
|  | 'PC_aa_C36_1' | -0.141 | 2.39E-01 | 3.84E-01 |
|  | 'PC_aa_C36_2' | -0.106 | 3.80E-01 | 5.10E-01 |
|  | 'PC_aa_C36_3' | -0.166 | 1.68E-01 | 2.93E-01 |
|  | 'PC_aa_C36_4' | -0.217 | 6.97E-02 | 1.79E-01 |
|  | 'PC_aa_C36_5' | -0.093 | 4.38E-01 | 5.63E-01 |
|  | 'PC_aa_C36_6' | -0.099 | 4.13E-01 | 5.39E-01 |
|  | 'PC_aa_C38_0' | -0.126 | 2.96E-01 | 4.45E-01 |
|  | 'PC_aa_C38_1' | -0.224 | 6.06E-02 | 1.70E-01 |
|  | 'PC_aa_C38_3' | -0.217 | 6.92E-02 | 1.79E-01 |
|  | 'PC_aa_C38_4' | -0.258 | 3.00E-02 | 1.59E-01 |
|  | 'PC_aa_C38_5' | -0.199 | 9.58E-02 | 2.04E-01 |
|  | 'PC_aa_C38_6' | -0.21 | 7.92E-02 | 1.88E-01 |
|  | 'PC_aa_C40_1' | -0.247 | 3.78E-02 | 1.59E-01 |
|  | 'PC_aa_C40_2' | -0.243 | 4.15E-02 | 1.59E-01 |
|  | 'PC_aa_C40_3' | -0.25 | 3.58E-02 | 1.59E-01 |
|  | 'PC_aa_C40_4' | -0.293 | 1.31E-02 | 1.59E-01 |
|  | 'PC_aa_C40_5' | -0.211 | 7.78E-02 | 1.88E-01 |
|  | 'PC_aa_C40_6' | -0.229 | 5.52E-02 | 1.60E-01 |
|  | 'PC_aa_C42_0' | -0.286 | 1.55E-02 | 1.59E-01 |
|  | 'PC_aa_C42_1' | -0.288 | 1.47E-02 | 1.59E-01 |
|  | 'PC_aa_C42_2' | -0.29 | 1.40E-02 | 1.59E-01 |
|  | 'PC_aa_C42_4' | -0.257 | 3.02E-02 | 1.59E-01 |
|  | 'PC_aa_C42_5' | -0.266 | 2.49E-02 | 1.59E-01 |
|  | 'PC_aa_C42_6' | -0.187 | 1.19E-01 | 2.32E-01 |
|  | 'PC_ae_C30_0' | -0.064 | 5.95E-01 | 6.86E-01 |
|  | 'PC_ae_C30_1' | -0.002 | 9.84E-01 | 9.84E-01 |
|  | 'PC_ae_C30_2' | -0.231 | 5.27E-02 | 1.60E-01 |
|  | 'PC_ae_C32_1' | -0.016 | 8.92E-01 | 9.13E-01 |
|  | 'PC_ae_C32_2' | -0.042 | 7.26E-01 | 7.96E-01 |
|  | 'PC_ae_C34_0' | -0.072 | 5.52E-01 | 6.54E-01 |
|  | 'PC_ae_C34_1' | -0.11 | 3.63E-01 | 5.10E-01 |
|  | 'PC_ae_C34_2' | 0.033 | 7.83E-01 | 8.49E-01 |
|  | 'PC_ae_C34_3' | 0.149 | 2.16E-01 | 3.60E-01 |
|  | 'PC_ae_C36_0' | -0.211 | 7.69E-02 | 1.88E-01 |
|  | 'PC_ae_C36_1' | -0.135 | 2.62E-01 | 4.06E-01 |
|  | 'PC_ae_C36_2' | -0.087 | 4.73E-01 | 5.86E-01 |
|  | 'PC_ae_C36_3' | 0.015 | 9.03E-01 | 9.13E-01 |
|  | 'PC_ae_C36_4' | -0.017 | 8.89E-01 | 9.13E-01 |
|  | 'PC_ae_C36_5' | -0.049 | 6.85E-01 | 7.61E-01 |
|  | 'PC_ae_C38_0' | -0.204 | 8.74E-02 | 1.92E-01 |
|  | 'PC_ae_C38_1' | -0.086 | 4.75E-01 | 5.86E-01 |
|  | 'PC_ae_C38_2' | -0.18 | 1.33E-01 | 2.45E-01 |
|  | 'PC_ae_C38_3' | -0.242 | 4.24E-02 | 1.59E-01 |
|  | 'PC_ae_C38_4' | -0.156 | 1.95E-01 | 3.32E-01 |
|  | 'PC_ae_C38_5' | -0.076 | 5.30E-01 | 6.36E-01 |
|  | 'PC_ae_C38_6' | -0.029 | 8.07E-01 | 8.55E-01 |
|  | 'PC_ae_C40_1' | -0.268 | 2.41E-02 | 1.59E-01 |
|  | 'PC_ae_C40_2' | -0.192 | 1.09E-01 | 2.22E-01 |
|  | 'PC_ae_C40_3' | -0.242 | 4.22E-02 | 1.59E-01 |
|  | 'PC_ae_C40_4' | -0.235 | 4.90E-02 | 1.60E-01 |
|  | 'PC_ae_C40_5' | -0.217 | 6.88E-02 | 1.79E-01 |
|  | 'PC_ae_C40_6' | -0.131 | 2.77E-01 | 4.22E-01 |
|  | 'PC_ae_C42_0' | -0.3 | 1.09E-02 | 1.59E-01 |
|  | 'PC_ae_C42_1' | -0.276 | 1.98E-02 | 1.59E-01 |
|  | 'PC_ae_C42_2' | -0.255 | 3.19E-02 | 1.59E-01 |
|  | 'PC_ae_C42_3' | -0.245 | 3.98E-02 | 1.59E-01 |
|  | 'PC_ae_C42_4' | -0.182 | 1.28E-01 | 2.44E-01 |
|  | 'PC_ae_C42_5' | -0.207 | 8.36E-02 | 1.92E-01 |
|  | 'PC_ae_C44_3' | -0.231 | 5.27E-02 | 1.60E-01 |
|  | 'PC_ae_C44_4' | -0.181 | 1.30E-01 | 2.44E-01 |
|  | 'PC_ae_C44_5' | -0.124 | 3.02E-01 | 4.46E-01 |
|  | 'PC_ae_C44_6' | -0.107 | 3.75E-01 | 5.10E-01 |
| 'Sphingolipids' | 'SM_OH_C14_1' | -0.014 | 9.09E-01 | 1.13E+00 |
|  | 'SM_OH_C16_1' | -0.077 | 5.25E-01 | 1.06E+00 |
|  | 'SM_OH_C22_1' | -0.045 | 7.07E-01 | 1.00E+00 |
|  | 'SM_OH_C22_2' | -0.066 | 5.83E-01 | 1.03E+00 |
|  | 'SM_OH_C24_1' | 0.013 | 9.17E-01 | 1.04E+00 |
|  | 'SM_C16_0' | -0.077 | 5.22E-01 | 1.23E+00 |
|  | 'SM_C16_1' | -0.108 | 3.68E-01 | 1.30E+00 |
|  | 'SM_C18_0' | -0.158 | 1.89E-01 | 1.00E+00 |
|  | 'SM_C18_1' | -0.165 | 1.68E-01 | 2.67E+00 |
|  | 'SM_C20_2' | -0.05 | 6.81E-01 | 1.07E+00 |
|  | 'SM_C24_0' | -0.084 | 4.86E-01 | 1.37E+00 |
|  | 'SM_C24_1' | -0.161 | 1.80E-01 | 1.43E+00 |
|  | 'SM_C26_1' | -0.007 | 9.53E-01 | 1.00E+00 |
| **'Other'** | 'H1' | -0.131 | 2.77E-01 | - |

Estimate are from univariate regression analyses within each metabolite family (i.e., acylcarnitine, amino acids, biogenic amines, glycerophospholipids and sphingolipids) by using false discovery rate (FDR) to take into account multiple comparisons.
